# Evaluation of holy basil mouthwash as an adjunctive plaque control agent in a four day plaque regrowth model

**DOI:** 10.4317/jced.51479

**Published:** 2014-12-01

**Authors:** Manasa Hosamane, Anirudh B. Acharya, Chhavi Vij, Dhiraj Trivedi, Swati B. Setty, Srinath L. Thakur

**Affiliations:** 1 Graduate student. Department of Periodontics, S.D.M. College of Dental Sciences and Hospital, Dharwad, Karnataka, India; 2 Professor. Department of Periodontics, S.D.M. College of Dental Sciences and Hospital, Dharwad, Karnataka, India; 3 Undergraduate student. Department of Periodontics, S.D.M. College of Dental Sciences and Hospital, Dharwad, Karnataka, India; 4 Professor and Head. Department of Biochemistry, S.D.M. College of Medical Sciences and Hospital, Dharwad, Karnataka, India; 5 Professor and Head. Department of Periodontics, S.D.M. College of Dental Sciences and Hospital, Dharwad, Karnataka, India; 6 Principal and Professor. Department of Periodontics, S.D.M. College of Dental Sciences and Hospital, Dharwad, Karnataka, India

## Abstract

Objectives: Various antibacterial and antiplaque agents are used in chemical plaque control but none are without their shortcomings. Chlorhexidine considered a gold standard, also has an array of side effects. To overcome these, numerous herbal extracts have been tried and tested and one among them is holy basil. The present study evaluated the antibacterial efficacy of holy basil in vitro against some periodontopathogens and its antiplaque effect in vivo. Study Design: Thirty periodontally healthy volunteers were randomly divided into three groups and refrained from all mechanical oral hygiene measures for 4 days and used one of the randomly assigned mouthwash (1- chlorhexidine; 2- holy basil; and 3- sterile water [placebo]) twice daily. The Plaque Index (PI) was assessed at days 0 and 5. Aqueous extract of holy basil was tested against Prevotella intermedia (P. intermedia) and Fusobacterium nucleatum (F.nucleatum). Results: Holy basil extract showed inhibition of both the tested periodontopathogens (P.intermedia and F.nucleatum) at various concentrations. In all groups, the PI increased from baseline to day 5. There was a statistically significant difference (p < .05) between the chlorhexidine and placebo rinse and the holy basil and placebo rinse, but no statistically significant difference was found between the chlorhexidine and holy basil rinse with respect to PI. Conclusions: These results indicate that the holy basil mouthwash has an antiplaque effect and is efficacious against P. intermedia and F. nucleatum strains in vitro. Hence holy basil mouthwash may have potential as an antiplaque mouthwash with prophylactic benefits.

** Key words:**Antibacterial agent, basil, Fusobacterium nucleatum, mouthwashes, Prevotella intermedia.

## Introduction

Ocimum sanctum commonly known as holy basil is an aromatic plant in the family Labiatae. The genus *Ocimum* includes aromatic herbs yielding essential oils, and has tropical distribution with nearly two-third of the 160 species reported from West Africa and the remaining one- third from Asia and America. India is represented by nine species of *Ocimum*, among which *Ocimum sanctum* is most prevalent (1). Holy basil is cultivated for religious and medicinal purposes, and for its essential oil.

The leaves of holy basil contain 0.7% volatile oil comprising about 71% eugenol and 20% methyl eugenol. The oil also contains carvacrol and sesquiterpine hydrocarbon caryophyllene. Fresh leaves and stem of holy basil extract yielded some phenolic compounds [antioxidants] such as cirsilineol, circimaritin, isothymusin, apigenin and rosameric acid, and appreciable quantities of eugenol. Two flavonoids, viz., orientin and vicenin from aqueous leaf extract of holy basil have been isolated. Ursolic acid, apigenin, luteolin, apigenin-7-O-glucuronide, luteolin-7-O glucuronide, orientin and molludistin have also been isolated from the leaf extract. Holy basil also contains a number of sesquiterpenes and monoterpenes viz., bornyl acetate, β -elemene, neral, camphene, campesterol, cholesterol and stigmasterol (2).

A variety of in vitro and in vivo studies have indicated some potential pharmacological properties of holy basil and its extracts. The anticancer activity of holy basil has been proved and cited by several investigators. The alcoholic extract of leaves of holy basil has a modulatory influence on carcinogen metabolizing enzymes such as cytochrome P 450, glutathione S-transferase [GST] etc., which are important in detoxification of carcinogens and mutagens. β-elemene, a constituent of holy basil has been studied for its potential anticancer properties, but human clinical trials have yet to confirm its effectiveness (3).

The antioxidant activity of holy basil has been reported by many workers and holy basil renders protection from radiation poisoning and can repair cells damaged by exposure to radiation (4). The fixed oil has demonstrated anti-hyperlipidemic and cardioprotective effects in rats fed a high fat diet (5). A double-blind trial conducted by Mondal S *et al.* [2011] (6) suggested that an alcoholic extract of holy basil modulates immunity, thus promoting immune system function. The essential oil of holy basil significantly possessed antiulcer activity due to its lipoxygenase inhibitory, histamine antagonistic and antisecretory effects (7).

Methanolic extract and aqueous suspension of holy basil showed analgesic, antipyretic and antiinflammatory effects in acute and chronic inflammations in rats (8) and according to a recent study holy basil acts a COX-2 inhibitor, proving its anti-inflammatory property (1). The other properties of holy basil includes, antibacterial property, hepatoprotective, antifertility, antiarthritic and anticoagulant activity (2).

Among all the properties of holy basil described, its anti-inflammatory action, bacteriostatic effect, antioxidant and immune modulatory properties make its use as a therapeutic agent for gingival and periodontal disease an appealing proposition. Hence, the aim of the present study was to evaluate the antibacterial and antiplaque efficacy of holy basil containing mouthwash in comparison with 0.2% chlorhexidine and sterile water as placebo.

## Material and Methods

- Study design:

The present study was carried out between January and February 2013 in this institution, and was divided into two parts. First, the aqueous extract of holy basil was prepared and antibacterial activity of holy basil extract was assessed by evaluating the minimum inhibitory concentration [MIC] against two periodontopathic organisms: *Fusobacterium nucleatum* [*F. nucleatum*], ATCC 25586 and *Prevotella intermedia* [*P. intermedia*] ATCC 25611. Second, a single-blinded, parallel design, clinical study was carried out and the 4-day plaque re-growth model was used to study the efficacy of the three mouthwashes.

- Test products:

The products used in the present study were [i] Holy basil mouthwash, [ii] Commercially available chlorhexidine [0.2%] mouthwash [Rexidin mouthwash, Indoco Remedies Ltd, Mumbai, India.] and [iii] Sterile water [Claris life sciences Ahmedabad, India.]

All of the three mouthwashes were dispensed in similar-looking opaque bottles.

- Preparation of holy basil mouthwash.

Preparation of aqueous extract of holy basil and preparation of the mouthwash was carried out in Department of Biochemistry, S.D.M. College of Medical Sciences and Hospital, Dharwad, Karnataka, India.

Dry leaves of holy basil were crushed to prepare a fine paste. Fifty grams of fine paste was added to 100 mL distilled water. The mixture of paste and distilled water were homogenized with a high speed blender. Care was taken to maintain the temperature below 150 C by providing cold water bath. One hundred mL distilled water was added and once again subjected to homogenization. The homogenized paste thus obtained was then filtered with muslin cloth by squeezing. Thirty three grams of remnant leaves paste was discarded. Two hundred and forty two ml of liquid extract was obtained after the above procedure. The liquid extract was then centrifuged at 5000 rpm for 10 min at 60 C to remove any suspended particles. The supernatant was collected in a sterile glass container and stored at refrigerated condition.

This aqueous extract contained 14 gm% extract from the paste and was considered as 100% extract for MIC activity. Further dilutions were prepared from the above 100% extract and MIC activity was analysed. For preparation of the mouthwash, distilled water was added to the 14 gm% aqueous extract to obtain 3.5gm% solution which was used to test the anti plaque efficacy in vivo.

- Evaluation of Minimum Inhibitory Concentration:

Minimum inhibitory concentration [MIC] is defined as the lowest concentration of an antimicrobial that will inhibit the visible growth of a microorganism after overnight incubation (9).

MIC of the holy basil extract was evaluated by broth dilution method against two periodontopathic organisms: *F. nucleatum* ATCC 25586 and *P. intermedia* ATCC 25611, to determine its antibacterial activity. Various dilutions of the holy basil extract [500 mg/mL, 250 mg/mL, 125 mg/mL, 62.5 mg/mL, 31.25 mg/mL,16.125 mg/mL, 8 mg/mL, 4 mg/mL, 2 mg/ mL, and 1 mg/mL] was tested against these organisms. Various dilutions of the holy basil extract and the organism to be tested was incubated in brain heart infusion agar for 24 hours and observed for turbidity.

- Clinical evaluation:

The design of the present study was in accordance with the Declaration of Helsinki and was approved by the Ethical Committee of this institution.

Thirty volunteers – 17 males and 13 females [age: 18 to 22 years] were recruited in the present study. These volunteers were students of this institution. Details of the present study were explained, and informed consent was obtained from the volunteers. Exclusion criteria include consumption of antibiotics or other medication in the last 3 months that might interfere with plaque formation, < 20 teeth to be included in the evaluation, presence of crowns or restorations, extensive bridges or prosthetic constructions and orthodontic appliances, known intolerance or allergy to mouthwashes, age below 18 years [18 years is the legal age in India to give informed consent without the permission of a parent/guardian] and pregnant women or lactating mothers.

On day 1, the Turesky *et al.* [1970] (10) modification of Quigley and Hein [1962] (11) Plaque Index using a 3% erythrosine dye [Agent P, ICPA Health Products Ltd, Mumbai, India.] was recorded. Using this index, plaque was assessed on the buccal/labial and lingual/palatal surfaces of all teeth.

The scoring criteria employed were as follows:

0 = No plaque.

1 = Separate flecks of plaque at the cervical margin of the tooth.

2 = A thin continuous band of plaque [up to 1 mm] at the cervical margin of the tooth.

3 = A band of plaque wider than 1 mm at the cervical margin of the tooth.

4 = Plaque covering at least one-third but less than two-thirds of the crown of the tooth.

5 = Plaque covering two-thirds or more of the crown of the tooth.

These indices were recorded by two trained and calibrated examiners [MHD and CV] who were blinded to the mouthwashes administered. Calibration was performed on three subjects, not included in the present study, and the ensuing scores were analysed by a third examiner. Scaling was performed by the same two calibrated examiners on all of the subjects recruited in the present study such that the areas stained with the disclosing agent were completely cleaned, to ensure zero plaque index scores. The baseline plaque index scores were calculated before scaling.

Volunteers were randomly allocated to one of the three groups, ten in each group. Group 1 [positive control] was the chlorhexidine group, Group 2 [test] was the holy basil group and Group 3 [negative control] was the sterile water group. For the following 4 test days, the volunteers had to refrain from carrying out all mechanical oral hygiene measures. Chewing gum was similarly not allowed. Instead, each volunteer rinsed for 1 min, twice daily [in the morning and evening after eating] with 10 ml of randomly allocated mouthwash solution.

The rinsing was monitored on all occasions by the examiners. The dietary regime of the patients was not altered, but it was ensured that the patients had a similar diet.

On day 5, the indices were re-recorded, and the volunteers were allowed to reinstate their routine oral hygiene procedures. These scores were the plaque re-growth scores which were subjected to statistical analysis.

## Statistical Evaluation

After the completion of re-recording of indices and decoding the mouthwash order, further evaluation was performed with the computer program Statistical Package for the Social Sciences [SPSS] version 11.0. The p value was set at ≤ 0.05. The mean plaque re-growth was calculated for each rinse solution. The normal distribution of the test data for each rinse solution and the differences compared to the placebo were checked with the Kolmogorov-Smirnov test and Shapiro Wilk test. Difference in the mean values between groups both at the base line and day 5 was determined by ANOVA followed by Tukey’s test for pair wise comparison. The paired t-test was used to test whether there was any significant difference between mean PI scores at baseline and day 5.

## Results

The in vitro study that was conducted for MIC evaluation showed that both the tested periodontopathogens [*P. intermedia* and *F. nucleatum*] were susceptible to holy basil extract at all the concentrations tested ranging from 10-1 to 10-9 suggesting a very high antibacterial activity.

Thirty subjects, 17 males and 13 females, completed the study. ANOVA [with *p* ≤ 0.05] showed no significant difference between mean of PI scores in the three groups at base line [*p* = 0.364]. In all the groups, PI showed significant increases from baseline to day 5 and there was a significant difference between mean of PI scores in the three groups on day 5 [*p* = 0.01]. The in vivo results showed that the mean difference in PI values from baseline to day 5 were highest for the placebo rinse [-0.8070] and the lowest for chlorhexidine [-0.2800]. The mean difference in PI value for holy basil [0.6880] was higher than chlorhexidine. The paired t test showed a statistically significant increase in the PI with holy basil [*p* = 0.04] and placebo [*p* = 0.01] as compared to chlorhexidine [*p* = 0.177] ( Table 1).

**Table 1:**
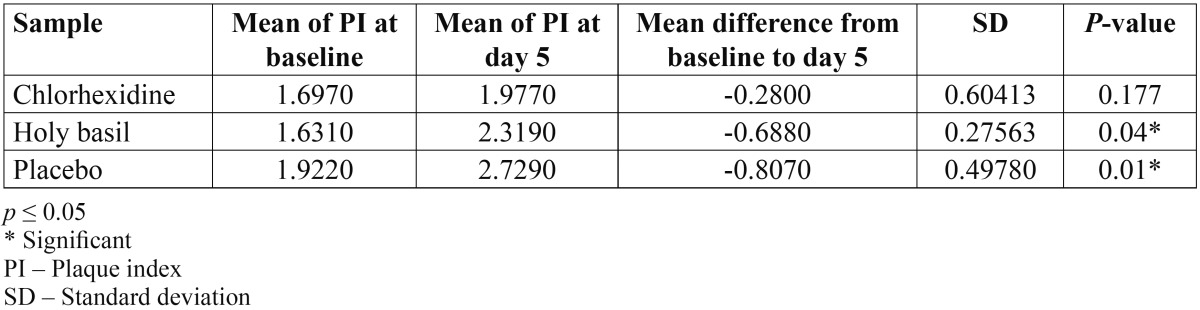
Results of in vivo plaque index comparing the mean PI scores of Baseline and Day 5 using paired t-test.

Differences between the individual rinse solutions and the placebo solution, determined via the Tukey Post hoc test, demonstrated significantly less plaque regrowth with respect to both chlorhexidine [*p* = 0.007] and holy basil [*p* = 0.041] as compared to the placebo. The difference between chlorhexidine and holy basil was not statistically significant [*p* = 0.187], suggesting the two rinses at the 95% confidence interval have a similar clinical efficacy.

## Discussion

Periodontal disease is a chronic inflammatory disease characterized by loss of connective tissue, alveolar bone resorption, and formation of periodontal pockets as a result of the complex interaction between pathogenic bacteria and the host’s immune response. Periodontitis starts as gingival inflammation mainly due to the presence of bacterial plaque, which, if left untreated, may progress and eventually lead to destruction of the entire periodontal attachment apparatus of the affected teeth (12). Prevention of periodontal disease should be based on the measures of supragingival plaque control thereby preventing gingivitis. Antibacterial measures such a good oral hygiene with or without chemotherapeutic agents, will diminish the plaque mass and reduce the injurious load on the tissues (13). Efficacy of mechanical plaque control in removing the bacterial plaque is well documented (14). But De La Rosa *et al* [1979] (15) showed that only half of the plaque was removed with brushing for 2 min. Rugg-Gunn and MacGregor [1978] (16) and MacGregor and Rugg-Gurm [1979] (17) suggested that certain tooth surfaces receive little or no attention during the brushing cycle. In combination with toothbrushing, daily use of the tested mouthwashes may result in a higher interproximal plaque reduction than daily flossing (18). Adjunctive use of chemicals would therefore appear to be a way of overcoming deficiencies in mechanical tooth cleaning habits. Among all of the anti-plaque and anti-gingivitis agents available, chlorhexidine continues to remain the ‘gold standard’ (19). However, it comes at the cost of several side effects. Hence, its long term use is not advocated. Excluding chlorhexidine containing rinses, only essential oil-containing rinse has been extensively evaluated and subsequently been shown to be of value as an adjunct to mechanical oral procedures (20,21). However, the alcohol content of essential oil rinses and its unpleasant taste are unacceptable to a few patients. Hence, the quest for a long-term, ideal and safe, anti-plaque and anti-gingivitis agent still continues.

The field of medicine is increasingly receptive to the use of antimicrobials derived from plant sources, as traditional antimicrobial agents become ineffective against both existing and new microbial diseases. The increasing problems of resistance to synthetic antimicrobials have encouraged the search for alternative natural products. Plants are the source of more than 25% of prescriptions and over-the-counter preparations, and the potential of natural agents for oral prophylaxis can therefore be considered (22).

The present short term study aimed to evaluate the antibacterial property of aqueous extract of holy basil against two periodonto pathogens in vitro and antiplaque efficacy in vivo. To evaluate the antiplaque efficacy, a four day plaque regrowth model was used. This study design can be described as an established method for testing the plaque inhibiting effect of an oral hygiene product as it used by numerous investigators (23-25). The advantage of four day plaque regrowth study design is that it eliminates the effect of adjunctive mechanical oral hygiene practices as they were not allowed during the study period. According to our knowledge, this is the first study to evaluate the antibacterial activity of holy basil against the periodonto pathogens [*P. intermedia* and *F. nucleatum*] in vitro and antiplaque efficacy in vivo.

Antimicrobial activity of holy basil extract was evaluated by various authors. Aqueous extract of holy basil showed growth inhibition for *Klesbiella, Escherecia coli, Proteus and Staphylococcus aureus* while alcoholic extract showed growth inhibition for *Vibrio cholerae*. The alcoholic extract was also found to be active against multidrug-resistant strains of *S. aureus* that are also resistant to common beta lactam antibiotics. Holy basil essential oil showed good antibacterial activity against *Bacillus pumilus, Pseudomonas aeruginosa* and *S. aureus* and higher content of linolenic acid in holy basil essential oil could contribute towards its antibacterial activity (26). In the dental literature, only one such study carried out by Agarwal *et al.* [2010] (26) is available in which antibacterial activity of various concentrations of holy basil extract was evaluated in vitro against *Streptococcus mutans* and they observed that at 4% concentration, holy basil showed the highest antibacterial activity.

The antioxidant activity of holy basil is one of the reasons for its widespread application in various fields of medicine. The antioxidant properties of flavonoids and their relation to membrane protection have been observed. Antioxidant activity of the flavonoids [orientin and vicenin] in vivo was expressed in a significant reduction in the radiation induced lipid peroxidation in mouse liver. Holy basil extract has significant ability to scavenge highly reactive free radicals. The phenolic compounds like cirsilineol, apigenin and rosmarinic acid, and eugenol from extract of fresh leaves and stems of holy basil possessed good antioxidant activity (27).

Methanolic extract and aqueous suspension of holy basil showed analgesic, antipyretic and antiinflammatory effects. To ascertain the inhibitory effect of holy basil essential oil on various mediators of inflammation, the antiinflammatory effect of the oil was evaluated against histamine, serotonin, bradykinin, and PGE2-induced paw edema in rats. The oil significantly inhibited edema formation induced by the inflammatory mediators. The fixed oil and linolenic acid possess significant antiinflammatory activity against PGE2, leukotriene and arachi-donic acid induced paw oedema in rats by virtue of their capacity to block both the cyclooxygenase and lipoxy-genase pathways of arachidonic acid metabolism. However essential oil was found to be devoid of analgesic activity in experimental pain models (2).

The results of the present study showed that holy basil-containing mouthwash was comparable to chlorhexidine with respect to its anti-plaque action with no statistically significant difference between the two.

The plaque inhibitory effect of holy basil mouth rinse can be attributable to its ability to kill dental plaque bacteria which can be correlated with the results of the in vitro study which comprised of evaluating antibacterial activity of holy basil extract against the two proven periodontopathogens *P. intermedia* and *F. nucleatum*. Results showed that both the organisms were sensitive to holy basil at all the concentrations tested [10 -1 to 10 -9 dilution], suggesting a potential antibacterial activity.

The limitations of the present study include the short time frame of the study, due to which the effect of the three mouthwashes on gingival inflammation could not be evaluated. Also, the parallel design of the present study could have affected the results, as every individual has a different rate of plaque growth. The anti-inflammatory and antioxidant action of holy basil has been cited in the literature. This was a coherent attempt to study its antibacterial and anti-plaque action. The potential strategies to harness these actions of holy basil could be supragingivally in the form of a mouthwash or a gel and subgingivally, in the form of irrigation or local drug delivery system.
